# Bifunctional *Aloe-Birch* Extracts for Green Silver Nanoparticle Synthesis and Synergistic Antimicrobial Microneedles Toward Infected Wound Healing

**DOI:** 10.3390/gels12080736

**Published:** 2026-08-17

**Authors:** Shun Zhang, Xu Liu, Yiyun Wei, Kun Bao, Xiaojuan Zhang, Chenlu Zhang

**Affiliations:** 1 School of Biological Science and Engineering, Shaanxi University of Technology, Hanzhong 723000, China; 15929874564@163.com (S.Z.); 15667909234@163.com (X.L.); 17764778099@163.com (Y.W.); bkc0822@163.com (K.B.);; 2 Shaanxi Province Key Laboratory of Bio-Resources, Hanzhong 723000, China; 3 Qinba State Key Laboratory of Biological Resources and Ecological Environment (Incubation), Hanzhong 723000, China

**Keywords:** green synthesis, silver nanoparticles, bioactive phytochemicals, hydrogel microneedles, infected wound healing

## Abstract

Plant-mediated green synthesis of silver nanoparticles (AgNPs) predominantly relies on single botanical sources, constraining reduction efficiency and forfeiting the therapeutic potential of residual biomass. This study employed aloe vera and birch bark extracts as reducing agents. A combination of single-factor analysis and response surface methodology was employed to prepare monodisperse 30 nm AgNPs (with a Zeta potential of −35.3 mV and confirmed by XPS as metallic Ag^0^). It was loaded into carboxymethyl chitosan-vanillin-polyvinyl alcohol (CVP) gel microneedles using the vacuum casting method. The mechanical strength of these microneedles reaches 0.302 N/needle, exceeding the skin penetration threshold. Throughout the entire preparation process, both its Ag^0^ chemical state and crystal structure were maintained (by FT-IR and XRD). The MIC values of AgNPs@CVP against four pathogens ranged from 0.977 to 7.813 μg/mL, with a biofilm disruption rate exceeding 70%, and demonstrated excellent biocompatibility (hemolytic rate < 1.6%, cell survival rate > 80%). In a rat wound infection model, high-dose microneedle therapy reduced the residual wound area to 10.9% by day 9. This strategy inhibited the secretion of TNF-α and IL-6 while promoting epithelial regeneration, angiogenesis, and collagen deposition. This bifunctional Aloe-Birch strategy converges green nanomaterial synthesis with therapeutic delivery within a single bio-based platform, advancing sustainable nanomedicine paradigms and providing a resource-efficient blueprint for clinical infectious wound management.

## 1. Introduction

The gel of *Aloe vera* is rich in anthraquinone glycosides, notably aloin and barbaloin, which exert broad-spectrum antimicrobial activity through microbial membrane disruption and reactive oxygen species generation, while concurrently promoting cell migration and collagen synthesis during wound healing [1]. *Birch bark* contains abundant pentacyclic triterpenes, primarily betulin and oleanolic acid, that demonstrate significant inhibitory effects against Gram-positive pathogens and suppress NF-κB-mediated inflammatory signaling through IκB kinase inhibition [2]. Leveraging these intrinsic bioactivities of medicinal plants, researchers have extensively explored green synthesis of silver nanoparticles (AgNPs) using single botanical extracts as both reducing and capping agents for infected wound therapy [3]. However, such single-source systems are inherently limited by constrained reduction efficiency, insufficient nanoparticle stability, and narrow antimicrobial spectra. Moreover, the residual biomass following extraction is typically discarded, representing incomplete valorization of the feedstock’s inherent bioactivity [4,5]. The synergistic employment of two medicinal plants with complementary antimicrobial mechanisms for AgNPs green synthesis holds promise for dual enhancement of reduction efficiency and bioactivity, yet the underlying synergistic reduction mechanism remains to be established.

Hydrogel microneedles represent an emerging transdermal drug delivery platform with significant advantages in the treatment of infectious wounds. This technology enables physical penetration of the biofilm barrier on wound surfaces to achieve localized high-concentration drug delivery, while simultaneously facilitating sustained drug release through the swelling properties of a three-dimensional cross-linked network, thereby minimizing systemic exposure risks [6,7]. Carboxymethyl chitosan (CMCS) exhibits excellent water solubility, biocompatibility, and gel-forming capability; the Schiff base cross-linked structure formed with Va enables the construction of an antibacterial three-dimensional network while avoiding the cytotoxicity issues associated with traditional glutaraldehyde cross-linking agents. The incorporation of polyvinyl alcohol (PVA) establishes a cross-linked polymer network through hydrogen bonding between PVA hydroxyl groups and CMCS amino groups, significantly enhancing the material’s mechanical strength and elastic recovery capacity.

Despite these material advances, the integration of AgNPs into hydrogel microneedles faces critical challenges. Free AgNPs are prone to aggregation under physiological electrolyte conditions, resulting in diminished antimicrobial activity and burst-release toxicity [8]. Although three-dimensional crosslinked networks theoretically enable uniform nanoparticle dispersion and stable loading, the interaction mechanisms between AgNPs and the CMCS-Va-PVA (CVP) gel matrix, the colloidal stability of embedded nanoparticles during and after fabrication, and their influence on microneedle mechanical performance and antimicrobial efficacy remain insufficiently elucidated. These gaps necessitate systematic investigation to realize the full potential of AgNPs-loaded hydrogel microneedles for infectious wound therapy.

**Figure 1:**
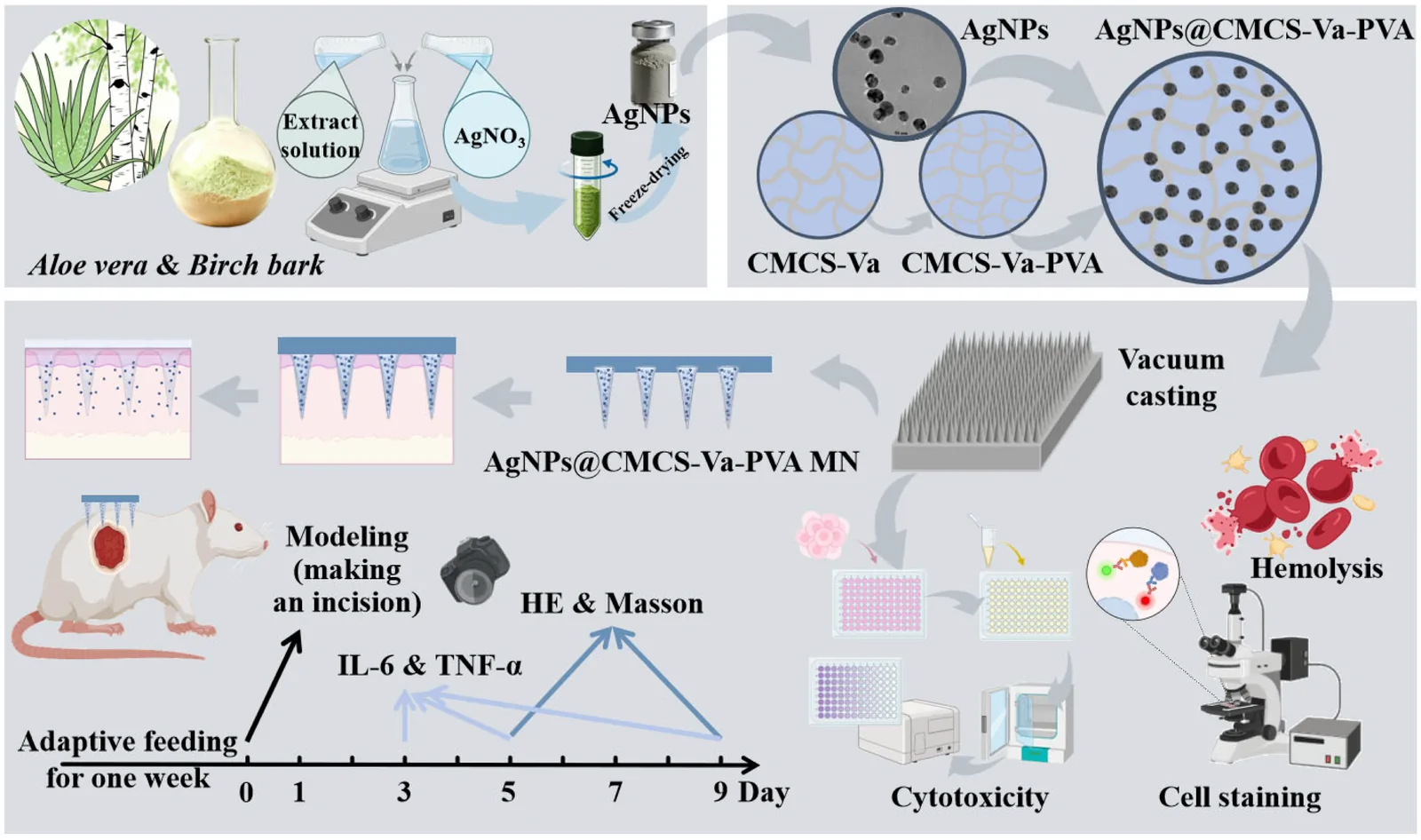
Green AgNPs synthesis, CVP microneedle loading, and triple synergistic wound healing.

Herein, the study proposes a strategy combining *Aloe vera* and *Birch bark* (AB) to synergistically green-reduce AgNPs and load them onto CVP cross-linked hydrogel microneedles, thereby establishing an integrated therapeutic platform that combines antibacterial, anti-inflammatory, and reparative effects (Figure 1). The synthesis process of AgNPs was optimized using RSM, and the morphology, structure, and chemical state of the nanoparticles were systematically characterized. Assess the mechanical properties, swelling behavior, and dispersion stability of AgNPs in microneedles; evaluate the in vitro antibacterial activity, biofilm removal capability, cellular compatibility, and blood compatibility of the system. Its healing-promoting efficacy was further validated in a rat model of full-thickness skin wounds infected with *S. aureus*, aiming to provide novel strategies for the green synthesis of nano-silver from dual medicinal ingredients and the treatment of infectious wounds.

## 2. Results and Discussion

### 2.1. Preparation and Characterization of Aloe Vera-Birch Bark Green Synthesis AgNPs

**Figure 2:**
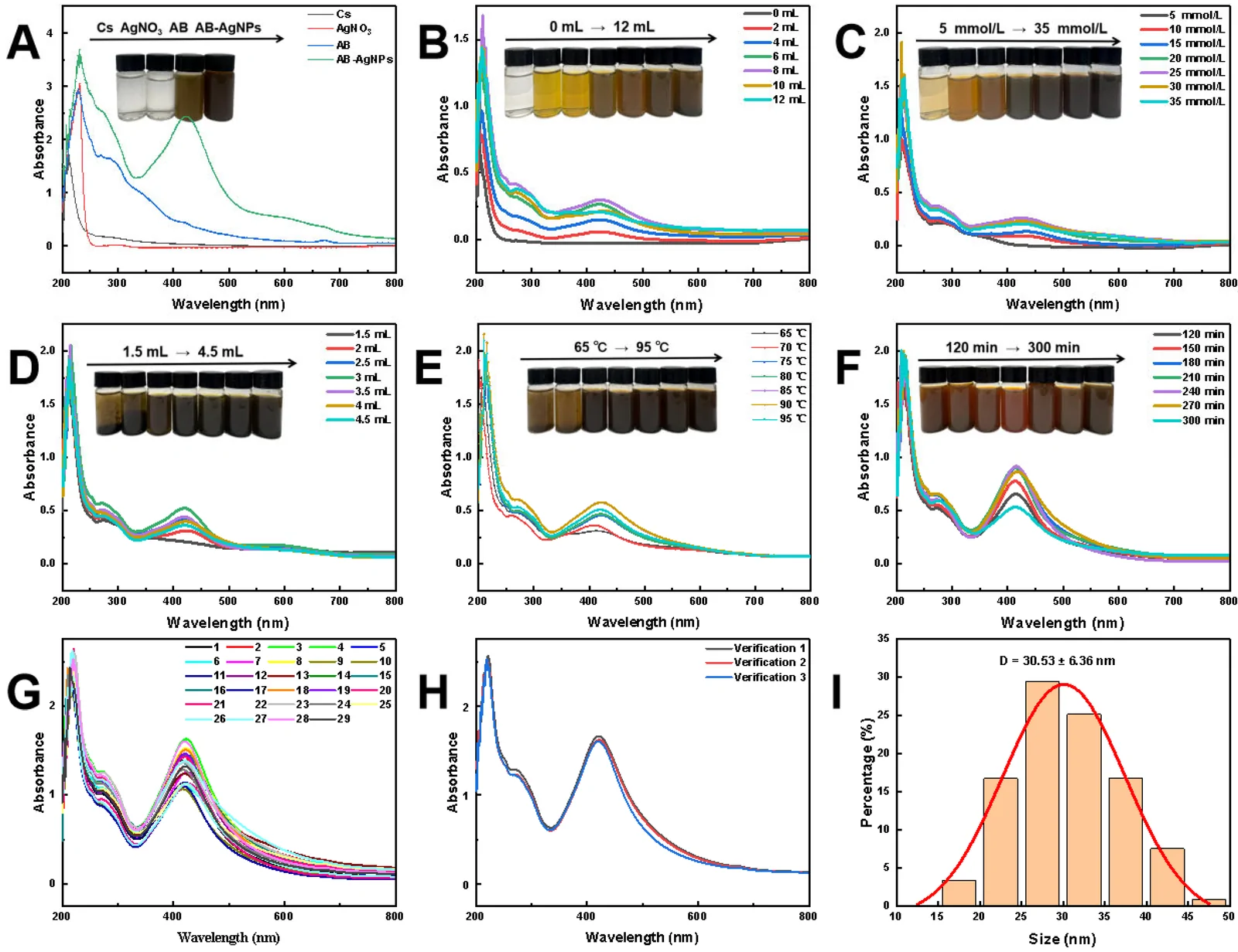
Optimization of AgNPs synthesis. (**A**) UV-Vis spectra confirming AgNPs formation; (**B**–**F**) single-factor optimization of extract dosage, AgNO_3_ concentration, CMCS dosage, temperature, and time; (**G**,**H**) RSM optimization and validation; (**I**) particle size distribution under optimal conditions.

To achieve efficient Ag^+^ reduction via *Aloe vera*-*birch bark* dual-botanical synergy, synthesis parameters were systematically optimized through single-factor experiments combined with RSM. UV-Vis analysis confirmed Ag^+^ reduction to metallic Ag^0^, with a characteristic SPR peak at 425 nm (Figure 2A). The symmetric peak profile and narrow FWHM indicated favorable monodispersity, while chitosan (Cs) incorporation enhanced colloidal stability through electrostatic stabilization [9].

Single-factor experiments investigated key parameters (Figure 2B–F): AgNO_3_ concentration (25.0 mmol/L ensured sufficient yield without aggregation), CMCS dosage (3.0 mL provided optimal dispersion stability), and reaction conditions (90 °C, 3.5 h furnished favorable kinetics). Box-Behnken design was subsequently employed for multi-parameter optimization (Supplementary Figure S1A, Table S1). Response surface methodology (RSM) substantially enhanced SPR peak intensity, improved symmetry, and reduced FWHM (Figure 2G) [8], demonstrating synergistic multi-parameter regulation outperformed single-factor adjustment. Validation experiments confirmed optimal conditions (8.0 mL extract, 25.4 mmol/L AgNO_3_, 3.0 mL Cs, 93 °C, 3.5 h) exhibited high consistency with model predictions (Figure 2H).

**Figure 3:**
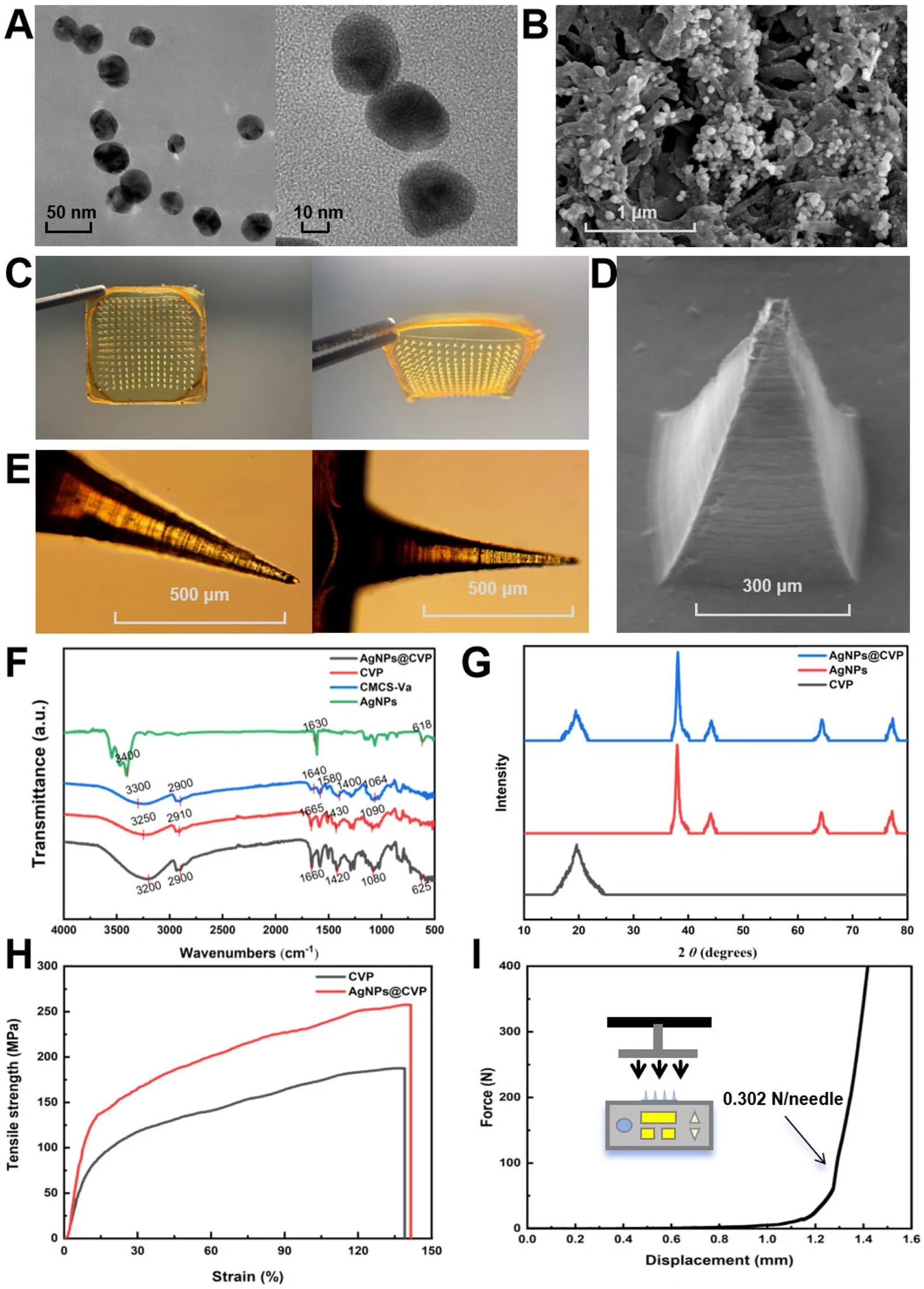
Characterization of AgNPs and AgNPs@CVP microneedles. (**A**,**B**) TEM and SEM of AgNPs; (**C**–**E**) macroscopic and microscopic morphology of microneedle patches; (**F**,**G**) FT-IR and XRD confirming Ag^0^ retention; (**H**,**I**) mechanical properties of gel microneedles.

TEM revealed uniform spherical morphology, smooth surfaces, and excellent dispersion without aggregation (Figure 3A). Particle size statistics indicated a narrow 25–35 nm distribution with a mean diameter of 30.05 nm (Figure 2I), critical for balancing antibacterial efficacy and biosafety [10,11]. SEM confirmed spherical morphology with a protective biomolecular coating derived from extract and Cs (Figure 3B). EDS mapping revealed uniform C, Ag, O, and Cl distribution (Supplementary Figure S1B,C, Table S2); homogeneous Ag signals confirmed excellent monodispersity, while trace Cl validated CMCS stabilization.

XPS determined elemental chemical states (Supplementary Figure S1D–F). The survey spectrum exhibited C 1s, Ag 3d, and O 1s peaks, confirming high purity. High-resolution C 1s deconvolution revealed three Gaussian peaks: 284.8 eV (C-C/C-H), 286.3 eV (C-O), and 288.9 eV (C=O), corresponding to aliphatic, hydroxyl/ether, and carbonyl groups from polysaccharides and polyphenols. Reducing moieties participated in nucleation, while stabilizing functionalities ensured long-term colloidal stability. High-resolution Ag 3d spectroscopy revealed characteristic spin-orbit doublets at 368.6 eV (Ag 3d_5/2_) and 374.7 eV (Ag 3d_3/2_) with a 6.1 eV splitting energy, unequivocally confirming metallic Ag^0^. Absence of silver oxide signals verified phase-pure metallic nanoparticles without oxidative by-products [12].

Collectively, these results confirm that the as-synthesized AgNPs possess a well-defined metallic core with a functionalized biomolecular shell.

### 2.2. Preparation and Morphological Characterization of AgNPs@CVP Microneedles

Nevertheless, free nanoparticles pose inherent risks such as aggregation and sudden release toxicity during in vivo applications; therefore, it is essential to design appropriate carrier systems to achieve stable encapsulation and delivery of nanoparticles. The material composition critically determines the formation efficiency and mechanical integrity of hydrogel microneedles [13]. As shown in Supplementary Figure S2A–C, under the optimized formulation (CMCS-Va: PVA = 1:1, *v*/*v*), the penetration efficiency of CVP gel microneedles was significantly higher than that of the CMCS-Va control group. AgNPs@CVP gel microneedles prepared under these optimal conditions were subsequently evaluated for both macroscopic and microscopic characteristics: digital imaging revealed a uniform light-yellow lamellar structure without phase separation or particle aggregation (Figure 3C), confirming that the CVP matrix successfully maintained and stabilized nanoparticle dispersion.

Optical microscopy revealed highly ordered microneedle arrays with sharp tips, distinct contours, and uniform height (800 μm), conforming to mold specifications for effective stratum corneum penetration (Figure 3E). SEM images demonstrated standard conical geometry with a small tip curvature radius, smooth dense surface morphology, and absence of pores or structural defects (Figure 3D). EDS elemental mapping showed uniform distribution of C, N, Na, O, and Ag throughout the matrix (Supplementary Figure S3A,B, Table S3). The homogeneous Ag signal distribution directly confirms that the AgNPs maintain a dispersed state without aggregation within the CVP cross-linked polymer network, a prerequisite for ensuring stable antibacterial efficacy and optimal biosafety. This uniform dispersion serves as a critical bridge linking AgNPs preparation to microneedle functionality [14]. The N signal originates from CMCS amide groups, while the Na signal arises from the CMCS-Va crosslinking byproduct; their coexistence corroborates the structural integrity of the CVP polymer network.

The XPS results further demonstrate excellent chemical compatibility between AgNPs and the CVP matrix (Supplementary Figure S3C–E). Spectral analysis reveals characteristic peaks at C 1s, N 1s, O 1s, and Na 1s in the CVP matrix, accompanied by a distinct Ag 3d signal. Compared to free AgNPs (368.6 eV and 374.7 eV), the binding energy is reduced by 0.7 eV, reflecting subtle chemical environment modifications induced by CVP matrix encapsulation. The unchanged peak shape confirms that the metallic state of Ag0 was fully preserved during the microneedle preparation process. Morphological and elemental analyses thus confirmed the structural integrity and uniform dispersion of AgNPs within the microneedles.

### 2.3. Structural and Performance Analysis of AgNPs and AgNPs@CVP

Functional realization depends not solely on physical encapsulation, but equally on molecular interactions, network stability, and mechanical compatibility. Systematic characterization of chemical structure, crystalline properties, and physical behavior is therefore imperative.

FT-IR elucidated molecular interactions within the hybrid system (Figure 3F). The O-H/N-H band at 3200–3500 cm^−1^ exhibited red-shifting with increased peak width, indicating strong intermolecular hydrogen bonds among CMCS, PVA, and Va. The C-H peak at 2900 cm^−1^ remained unshifted, confirming polymer backbone retention. The emergence of a new peak at 1665 cm^−1^, along with the disappearance of the Va aldehyde peak, verified Schiff base C=N bond formation. Attenuated intensity at 1420 cm^−1^ indicated carboxyl participation in hydrogen bonding. C-O-C and C-O vibrations remained stable at 1080–1090 cm^−1^, demonstrating that AgNPs introduction did not disrupt the backbone [15]. Critically, extract peaks at 618–625 cm^−1^ were retained without Ag-O signatures, confirming physical encapsulation, corroborated by XPS evidence of preserved Ag^0^ state. The hybrid system thus forms a homogeneous structure through hydrogen bonding and Schiff base interactions.

XRD confirmed crystal structure and stability (Figure 3G). AgNPs exhibited peaks at 2θ = 38.1°, 44.3°, 64.5°, and 77.5°, corresponding to fcc metallic silver (JCPDS No. 04-0783). AgNPs@CVP retained all Ag^0^ peaks with slight intensity attenuation, without broadening or shifting, confirming crystalline integrity throughout fabrication [14]. Zeta potential (−35.3 mV, Supplementary Figure S3F) and UV-Vis spectroscopy validated colloidal stability. The 420 nm SPR peak was retained without broadening after CVP incorporation (Supplementary Figure S3G), confirming uniform dispersion and intact functional activity.

Tensile testing revealed a fracture strength of 257 MPa and an elongation of 141.5%, a 37.1% improvement over pure CVP gel (Figure 3H). Nano-reinforcement arises from uniformly dispersed AgNPs as stress transfer centers. Microneedle compression yielded 0.302 N/needle (Figure 3I), substantially exceeding the 0.058 N/needle penetration threshold [16,17]. Swelling analysis demonstrated rapid water absorption with maintained structural integrity (Supplementary Figure S3H), facilitating gradual silver release for acute infection control and prolonged antibacterial activity, integrating physical penetration with drug-release kinetics.

This study employed a synergistic combination of AB extracts to facilitate the green synthesis of AgNPs, successfully yielding nanomaterials with uniform particle size (30 ± 5 nm) and stable Zeta potential (−30 mV). These results represent a significant breakthrough in the field of controlled synthesis of nanomedicines. While traditional single-plant extraction methods eliminate the use of harmful chemical reagents, they often suffer from challenges such as uneven reduction capacity, particle aggregation, or insufficient stability [12,18]. The dual-plant system employed in this study not only enables efficient synthesis of AgNPs but also, through the synergistic interaction between aloe polysaccharides and the triterpenoids and phenolic acids abundant in birch bark, preserves a wealth of bioactive components, thereby endowing the nanomaterials with additional anti-inflammatory and antioxidant potential at the source [2,19]. Compared to conventional green synthesis methods that solely utilize *Aloe vera* or single herbal extracts [1,20], this dual-source driving mechanism effectively addresses the significant batch-to-batch variations observed when using a single biomass reductant in large-scale production. A Zeta potential of −30 mV indicates that the particle surface possesses sufficient electrostatic repulsion to maintain colloidal stability, which is highly consistent with recent research trends on how mixed plant extracts enhance the functionality and stability of nanomaterials [21]. Furthermore, this particle size range (30 ± 5 nm) is widely recognized as the optimal interval for balancing antibacterial efficacy and cytotoxicity, ensuring a large specific surface area for silver ion release while avoiding the nonspecific cellular damage associated with excessively small particle sizes [8]. Therefore, this dual-plant synergistic synthesis strategy provides a novel paradigm for developing highly stable and biocompatible nanobased antibacterial agents, significantly outperforming traditional single-step chemical reduction methods or standalone biosynthesis approaches.

### 2.4. Evaluation of Cellular Compatibility and Migration Capacity

Nonetheless, the paramount requirement for wound-treatment materials is balancing biocompatibility with functional activity, as high antimicrobial efficacy often entails cytotoxic risks. Systematic cellular-level evaluation is therefore imperative to establish the safety rationale for subsequent animal studies.

**Figure 4:**
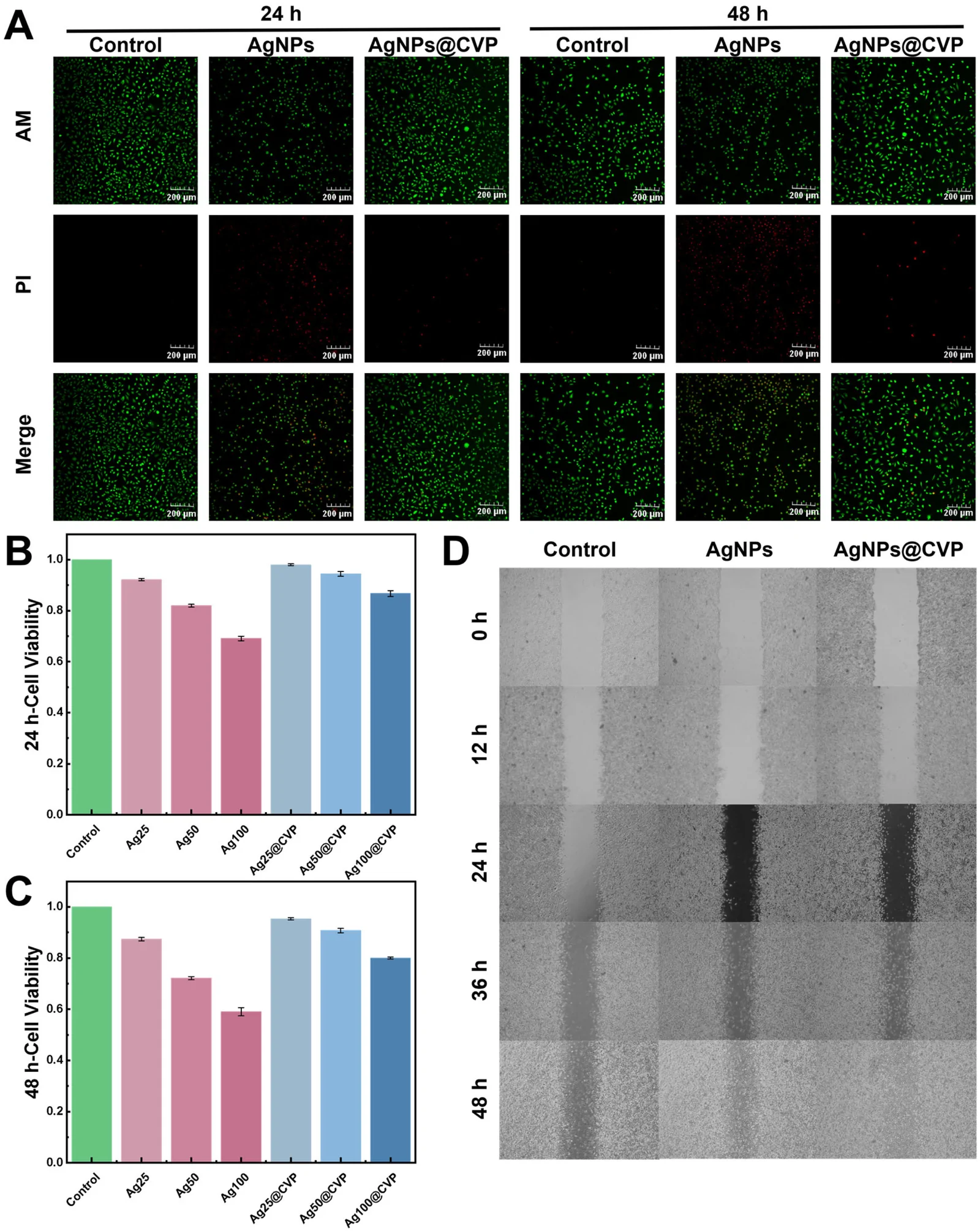
Biocompatibility and cell function evaluation. (**A**) Live/dead staining of L929 cells; (**B**,**C**) CCK-8 cytotoxicity assay at 24 and 48 h; (**D**) scratch assay evaluating fibroblast migration.

The CCK-8 assay quantified cytotoxicity using L929 mouse fibroblasts (Figure 4B,C). After 24 and 48 h, cell viability in the AgNPs@CVP group was significantly higher than in the free AgNPs group at all concentrations. The rate of viability decline markedly slowed with increasing concentration, indicating a protective effect of the CVP matrix. At 25, 50, and 100 μg/mL, AgNPs@CVP maintained viability exceeding 80% at 48 h, confirming low cytotoxicity. The 100 μg/mL safety threshold guided dose selection for animal studies.

Live/dead staining corroborated these findings (Figure 4A). At both 24 and 48 h, AgNPs@CVP exhibited substantially higher viable cell proportions and lower dead cell proportions compared with free AgNPs. The fluorescence distribution closely matched the blank control, demonstrating that the CVP matrix not only attenuates but effectively eliminates cytotoxic effects, achieving functional retention with reduced toxicity.

The scratch assay evaluated fibroblast migratory capacity (Figure 4D). AgNPs@CVP significantly accelerated wound closure over 48 h compared with both the blank control and the free AgNPs group. This enhanced migration is mediated by a dual mechanism: the CVP matrix mitigates AgNPs cytotoxicity while establishing a bioactive microenvironment conducive to cell migration.

Collectively, these experiments confirm that AgNPs@CVP achieves an optimized balance between safety and functionality, establishing 100 μg/mL as the safe upper dose limit and providing preliminary validation of the gradual release strategy.

### 2.5. Antimicrobial Performance and Mechanism of Action

**Figure 5:**
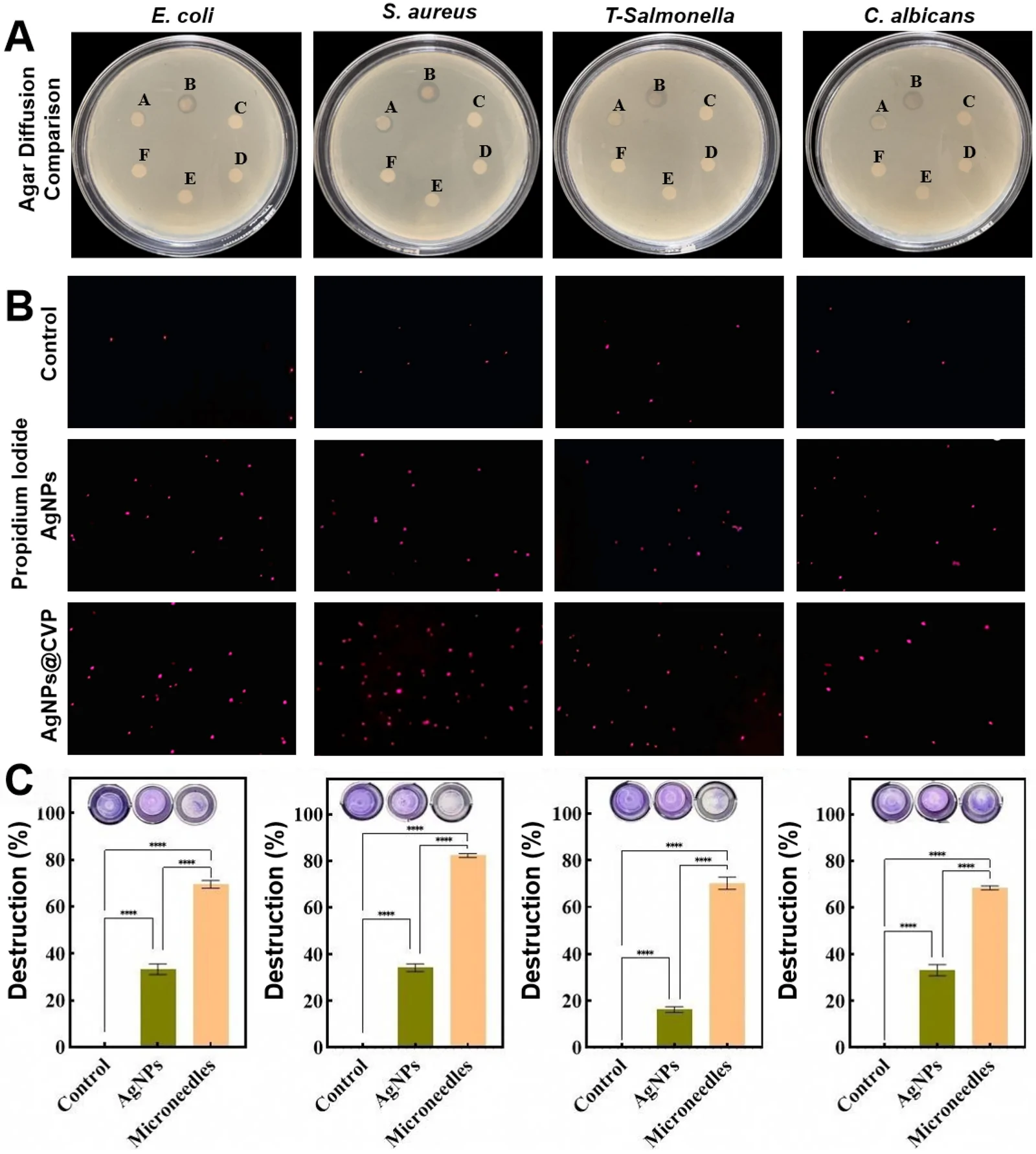
Antibacterial performance and mechanisms. (**A**) Agar diffusion tests against four pathogens; (**B**) PI staining indicating membrane damage; (**C**) biofilm inhibition rates. (A: AgNO_3_, B: AgNPs@CVP, C: CMCS, D: Va, E: PVA, F: control; ****, *p* < 0.0001).

A systematic evaluation was conducted on antibacterial efficacy and mechanisms of action against common clinical pathogens. Agar diffusion tests confirmed broad-spectrum activity (Figure 5A). Both AgNPs and AgNPs@CVP formed inhibition zones against all four pathogens, whereas CVP, CMCS, Va, and PVA alone showed no activity, confirming the effect was exclusively attributable to AgNPs. Under identical silver concentrations, AgNPs@CVP produced larger, more uniform inhibition zones than free AgNPs, attributed to localized AgNPs enrichment at the culture medium-gel interface prolonging antibacterial contact.

Quantitative MIC determination validated synergistic enhancement (Supplementary Figure S4A, Table S4). Free AgNPs exhibited MIC values of 23.438–31.25 μg/mL, whereas AgNPs@CVP achieved 0.977–7.813 μg/mL, corresponding to 3- to 32-fold improvement. The MIC of 0.977 μg/mL against *S. aureus* is more than 100-fold below the 100 μg/mL cellular safety threshold, providing robust evidence for therapeutic efficacy.

MBC assays confirmed potent bactericidal efficacy (Supplementary Figure S4B). AgNPs@CVP maintained narrow MBC/MIC ratios of 1.0–2.0 across all strains, indicating bactericidal rather than bacteriostatic activity crucial for acute infection control. This rapid sterilization correlates with the 30 nm particle size and high specific surface area. Time-dependent colony counts demonstrated potent bactericidal kinetics (Supplementary Figure S5), with CFUs declining significantly as exposure increased, confirming the CVP matrix sustains effective antimicrobial concentrations for complete pathogen eradication.

PI membrane permeability assays and biofilm inhibition experiments elucidated antimicrobial mechanisms. PI staining (Figure 5B) revealed minimal fluorescence in controls, indicating intact membranes. Free AgNPs induced detectable damage, whereas AgNPs@CVP generated significantly higher, more densely distributed PI signals. This enhancement is attributed to localized AgNPs enrichment improving microbial contact efficiency, with cationic CMCS contributing to electrostatic membrane disruption.

Biofilm inhibition experiments demonstrated striking differences (Figure 5C). Free AgNPs achieved only 20–40% inhibition, failing to penetrate the extracellular polysaccharide matrix. In contrast, AgNPs@CVP consistently exceeded 70% disruption across all pathogens, with nearly 80% against S. aureus. This superior activity stems from the three-dimensional cross-linked CVP network, which prolongs AgNPs exposure and enhances interaction with biofilm-encapsulated pathogens.

These studies establish a favorable therapeutic window, combining low MIC with high safety.

The loading of AgNPs onto CVP gel microneedles demonstrated the decisive influence of the matrix material on nanomedicine delivery efficiency, with reduced MIC values and a narrowed MBC/MIC ratio confirming a significant synergistic antibacterial effect. Existing literature indicates that although hydrogel dressings alone can maintain a moist environment, their antibacterial efficacy is limited, whereas free AgNPs are prone to exhibit reduced activity due to aggregation [22,23]. In this study, CMCS, as a natural cationic polysaccharide, not only exhibits excellent biocompatibility but also enables its amino and carboxyl groups on the molecular chain to form stable coordination bonds with AgNPs, thereby preventing particle aggregation [15,24]. As a natural crosslinking agent, Va not only confers excellent mechanical properties to hydrogels, but its inherent aldehyde group structure can also interact with the surface of AgNPs, modulating the sustained release kinetics of silver ions and thereby achieving more prolonged antibacterial efficacy [25,26]. Compared to free AgNPs, this composite system ensures high dispersion and sustained release of AgNPs at the wound interface through the confinement effect of the polymer network. This finding aligns with recent reports on how chitosan/PVA-based hydrogels enhance the stability of AgNPs [14,27]. The narrowed MBC/MIC ratio indicates that this composite material exhibits stronger bactericidal rather than merely bacteriostatic properties, which is crucial for eliminating deep tissue infections. This synergistic effect not only enhances antibacterial efficacy but also reduces the total dosage of silver ions required, thereby potentially lowering the risk of heavy metal accumulation during long-term application and highlighting the unique advantages of material design in optimizing drug-release profiles [28,29].

In terms of biofilm removal efficacy, this composite hydrogel demonstrated a biofilm disruption rate exceeding 70%, significantly surpassing the typical 20–40% achieved by free AgNPs. These results represent a major advancement in strategies for combating antibiotic-resistant bacterial biofilms. Bacterial biofilms pose a significant barrier to chronic wound healing, as their extracellular polysaccharide matrix often impedes the penetration of antimicrobial agents, leading to failure of conventional antibiotic therapy [30,31]. In this study, the positive charge property of CMCS facilitates disruption of negatively charged biofilm matrices through electrostatic interactions, while the PVA network enhances the deep penetration of AgNPs. This dual mechanism combining physical disruption and chemical eradication explains its outstanding anti-biofilm efficacy [32,33]. Meanwhile, the low hemolytic rate of 1.6% and a cell survival rate exceeding 80% demonstrate the excellent biosafety of this material, overcoming the potential cytotoxicity associated with traditional high-concentration silver preparations [34]. This result is highly consistent with recent research objectives regarding nano-silver composite dressings that maintain high antimicrobial efficacy while reducing host toxicity. Numerous studies have demonstrated that high concentrations of free silver ions exert toxic effects on fibroblasts and keratinocytes, thereby delaying the healing process. In contrast, the composite system in this study effectively mitigates this risk through a sustained-release mechanism [35]. Therefore, this material not only exhibits strong resistance to biofilms but also maintains excellent biocompatibility, providing a safe and effective solution for addressing clinical infections caused by multidrug-resistant bacteria—a feature of significant clinical relevance amid the increasingly severe challenge of antimicrobial resistance (AMR) [36].

### 2.6. Hemolytic Compatibility and Animal Wound Healing Experiment

Nevertheless, in vitro antibacterial performance does not guarantee in vivo therapeutic efficacy, as interactions with blood and tissue components may alter both safety and effectiveness. Hemocompatibility evaluation and animal model validation are therefore essential bridges toward clinical translation.

Hemolysis assays confirmed excellent blood compatibility (Supplementary Figure S6A,B). The AgNPs@CVP group (100 μg/mL) exhibited a hemolysis rate of merely 1.6%, substantially below the 5% threshold for clinical biomaterials. Free AgNPs at equivalent concentration showed a higher rate of 2.3%, within the safety range yet significantly exceeding the encapsulated formulation. These data establish a robust safety profile supporting clinical translation.

**Figure 6:**
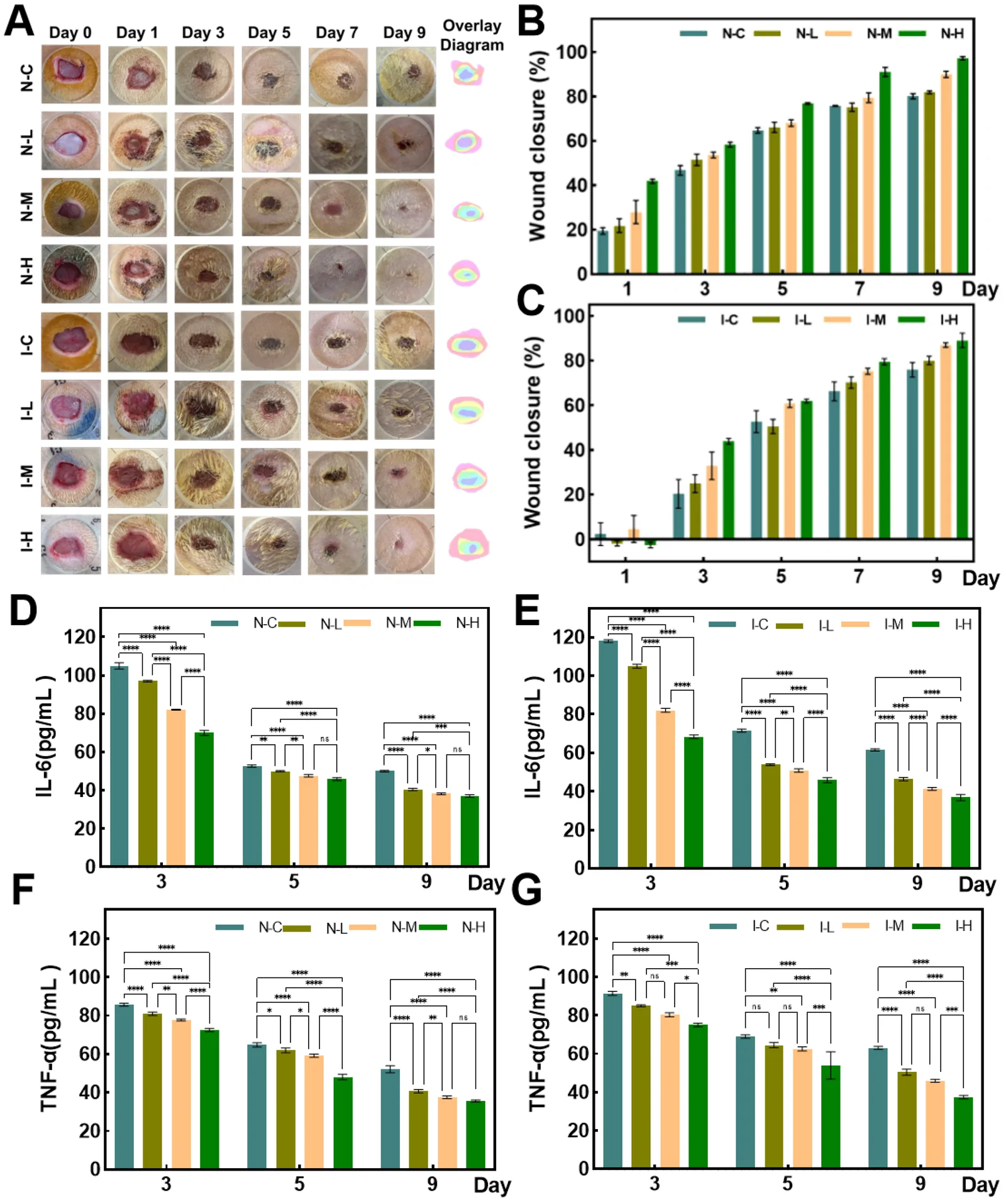
Wound healing and inflammatory cytokine analysis. (**A**) Representative wound images; (**B**,**C**) wound closure rates over 9 days; (**D**–**G**) TNF-α and IL-6 levels in normal and infected wounds. (*, *p* < 0.1; **, *p* < 0.01; ***, *p* < 0.001; ****, *p* < 0.0001; ns, no significance).

Therapeutic performance was evaluated in a rat full-thickness wound model comprising normal (N-) and *S. aureus*-infected (I-) wounds. Macroscopic evaluation revealed marked intergroup differences (Figure 6A). In normal wounds, the NH group demonstrated reduced exudate by day 1, pronounced granulation by day 3, and near-complete healing by day 9, correlating with rapid bactericidal activity that promptly controls microbial burden. Conversely, the NC group exhibited substantially delayed healing with considerable unhealed tissue persisting through day 9. In infected wounds, the IH group effectively suppressed infection spread by day 3, induced significant wound contraction by day 5, and achieved superior healing rates by day 9.

Quantitative wound area analysis confirmed dose-dependent efficacy (Figure 6B,C). In normal wounds, the NH group achieved 2.78% residual area at day 9 versus 19.87% for NC. In infected wounds, the IH group reached 10.93% versus 24.10% for IC.

Enzyme-linked immunosorbent assay (ELISA) quantification of TNF-α and IL-6 revealed distinct cytokine kinetics (Figure 6D–G). In normal wounds, AgNPs@CVP induced a transient elevation during days 1–3, followed by a rapid decline to near baseline by day 5, reflecting productive short-term inflammatory activation with timely resolution. In contrast, the IC group exhibited persistently elevated cytokines throughout the observation period, driving pathological hyperinflammation. The IH group significantly suppressed pro-inflammatory expression as early as day 3, demonstrating effective inflammatory regulation.

These animal experiments confirm that AgNPs@CVP microneedles achieve excellent blood safety, infection control, and inflammatory modulation, successfully translating in vitro findings to in vivo efficacy.

### 2.7. Histological Evaluation of Tissue Regeneration and Collagen Deposition

**Figure 7:**
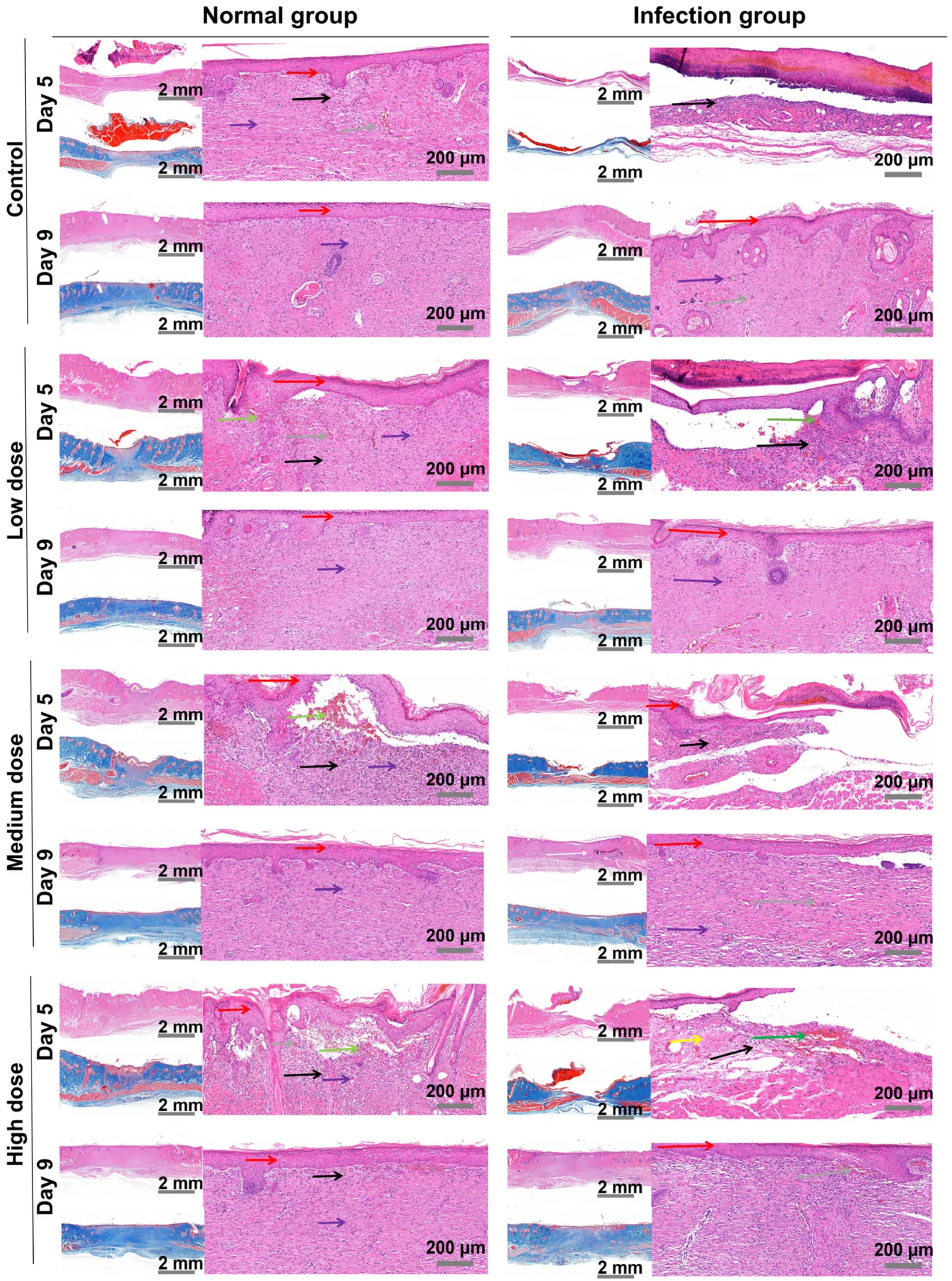
H&E and Masson staining analysis of wound healing process in rats. (Red arrow: epidermal hyperplasia; Black arrow: inflammatory cell infiltration; Green arrow: bleeding; Yellow arrow: edema; Gray arrow: capillary proliferation).

However, macroscopic healing improvements require histological corroboration, as tissue structural reconstruction quality remains a critical criterion for wound healing assessment. Key reparative events, including epidermal barrier restoration, collagen deposition, and angiogenesis, were therefore validated through histopathological examination. Histopathological examination of wound biopsies from normal and infected rat models at days 5 and 9 assessed re-epithelialization, inflammatory infiltration, hemorrhage, edema, angiogenesis, and collagen deposition via H&E and Masson staining (Figure 7).

At day 5, the NH group exhibited significant epidermal proliferation, active angiogenesis, and moderate inflammatory infiltration with minor hemorrhage and edema, a proliferative-dominant phenotype corroborating scratch assay results. The NC group showed extensive inflammatory infiltration, severe edema, delayed epidermal regeneration, and sparse neovascularization, reflecting persistently elevated TNF-α/IL-6 levels. By day 9, NH achieved complete epidermal barrier repair with orderly collagen fibers and capillary density approaching normal skin.

Under infectious conditions, differences were more pronounced. At day 5, the IC group exhibited severe diffuse inflammatory infiltration, extensive hemorrhage, significant edema, impaired epidermal proliferation, vascular defects, and focal necrosis, the pathological hallmark of an infection-inflammation vicious cycle. In contrast, AgNPs@CVP microneedles effectively restricted inflammatory spread, reduced infiltration, and induced epidermal proliferation with neovascularization. This concurrent infection control and structural reconstruction provides direct histological evidence for the triple synergistic mechanism, validating that >70% biofilm disruption translates to reduced inflammation in vivo. By day 9, the IH group formed a mature stratified epidermal barrier with dense granulation tissue, uniform collagen deposition, well-developed microvascular networks, and resolved inflammation-establishing a structure-function correlation with the 10.93% residual wound area. The IC group maintained persistent inflammation with epidermal discontinuities, sparse angiogenesis, and delayed repair.

These findings confirm that AgNPs@CVP microneedles accelerate wound transition from inflammatory through proliferative and remodeling phases via three synergistic mechanisms: suppression of excessive inflammation, promotion of epidermal proliferation and barrier reconstruction, and enhancement of angiogenesis and collagen deposition. This microscopic-macroscopic consistency provides comprehensive histopathological validation for the integrated antibacterial, anti-inflammatory, and repair-promoting therapeutic mechanism.

In vivo experimental data demonstrate that in infection wound models, the remaining wound area treated with this composite hydrogel on day 9 was only 10.93%, significantly lower than the 24.10% observed in the control group; this healing rate surpasses that reported for most similar silver-loaded dressings. In analogous infection or diabetic wound models, traditional silver dressings or single hydrogels typically exhibit lower wound closure rates at comparable time points and often require longer durations to achieve equivalent healing outcomes [28]. This outstanding therapeutic efficacy is attributed to the moist healing environment provided by the hydrogel, the potent anti-infective properties of AgNPs, and the bioactive effects of CMCS in promoting fibroblast migration and collagen deposition [15,23]. Compared to single-function dressings that rely solely on antibacterial effects [29], the multifunctional system developed in this study achieves simultaneous advancement of anti-infection and tissue regeneration. The moist environment facilitates cell migration and the activity of growth factors, while the potent antibacterial effect of AgNPs creates a sterile microenvironment conducive to tissue regeneration [8]. Furthermore, the inherent healing-promoting properties of CMCS further accelerate the epithelialization process [24]. This discovery not only validates the application potential of this material in complex infected wounds, but also provides crucial experimental evidence and theoretical support for developing next-generation smart-responsive wound dressings [36]. Compared with other silver nanocomposite dressings reported in the existing literature [37], the AgNPs@CVP system in this study achieves a superior balance between healing speed and safety, demonstrating significant potential for clinical translation.

## 3. Conclusions

This study established an integrated platform combining two plant components, AB, to achieve green reduction of silver nanoparticles (AgNPs) through synergistic effects, utilizing a CVP cross-linked polymer network hydrogel microneedle delivery system. The dual-plant extracts serve as reducing agents, effectively overcoming the key limitations inherent in green synthesis methods based on single sources. AgNPs with a monodispersity of 30 nm and excellent colloidal stability (Zeta potential −35.3 mV) were prepared using the RSM; XPS analysis confirmed their metallic Ag^0^ state without oxidative by-products, thereby avoiding the use of hazardous reagents associated with conventional approaches. The AgNPs@CVP microneedles achieved mechanical strength of 0.302 N/needle, exceeding the threshold for skin penetration. The integrated system exhibited 3- to 32-fold enhancement in antibacterial efficacy (MIC 0.977–7.813 μg/mL) with narrow MBC/MIC ratios (1.0–2.0), and biofilm disruption exceeding 70%. A concurrent favorable safety profile, with hemolysis of 1.6% and cell viability >80%, established a >100-fold therapeutic window. In rat *S. aureus*-infected wound models, high-dose microneedles reduced residual wound area to 10.93% by day 9 versus 24.10% in controls. Transient inflammatory cytokine resolution and histologically confirmed re-epithelialization, angiogenesis, and collagen deposition demonstrate active wound modulation through antibacterial, anti-inflammatory, and pro-reparative triple synergy. This enhanced therapeutic mechanism integrates sustainable nanomaterial synthesis with drug delivery functionality into a single bio-based platform, advancing the development paradigm of green nanomedicine in infectious wound treatment.

## 4. Materials and Methods

### 4.1. Materials and Reagents

*Aloe vera* was purchased from Hanzhong City, Shaanxi Province, while *birch bark* was collected from the Greater Khingan Range in Heilongjiang Province. Both raw materials were authenticated by Professor Chenlu Zhang from the School of Biological Science and Engineering, Shaanxi University of Technology, then pulverized and sieved through a 50-mesh sieve for subsequent experiments. Carboxymethyl chitosan (CMCS), Va, polyvinyl alcohol (PVA), Cs, and AgNO_3_ were of analytical grade and used without further purification. The L929 mouse fibroblast cell line was purchased from Procell Life Science & Technology Co., Ltd., Wuhan, China.

### 4.2. Green Synthesis, Process Optimization and Characterization of AgNPs

AB extract was obtained using a standard water bath extraction protocol [38], and a water-based Cs acetate solution was separately prepared as a nanoparticle stabilizer. In the preliminary synthesis of AgNPs, 5 mL of AB extract was first placed in a 50 mL brown conical flask to avoid light-induced degradation. Subsequently, 3 mL of Cs acetate solution and 10 mL of 20 mmol/L AgNO_3_ aqueous solution were added sequentially, followed by constant magnetic stirring at 70 °C for 12 h to promote reduction and stabilization reactions [9]. Following synthesis, the resulting AgNPs suspension was vacuum-filtered to remove any unreacted residues and then stored at 4 °C in the dark until further characterization and use.

Single-factor optimization experiments were designed to investigate the effects of key parameters (AB extract dosage, AgNO_3_ concentration, Cs dosage, reaction temperature, and reaction time) on the synthesis efficiency and stability of AgNPs. Based on the single-factor results, RSM was further adopted for the coupled optimization of critical parameters to determine the optimal preparation process for AgNPs with high dispersion and stable performance.

The morphological characteristics of AgNPs were characterized using transmission electron microscopy (TEM, SU8010, Hitachi, Tokyo, Japan) and scanning electron microscopy (SEM, EVO15, Zeiss, Oberkochen, Germany). Elemental composition and distribution were analyzed by energy dispersive spectrometer (EDS, UltimMax170, Oxford Instruments, Abingdon, UK). The crystal structure of the samples was determined using an X-ray diffractometer (XRD, D8 Advance, Bruker, Karlsruhe, Germany) with copper target Kα radiation (40 kV, 40 mA, 2θ scanning range 10–90°, step rate 0.017/s). X-ray photoelectron spectroscopy (XPS, Thermo Fisher Scientific Escalab Xi+, Thermo Fisher Scientific, East Grinstead, UK) was employed to analyze the chemical states of elements.

### 4.3. Preparation and Characterization of AgNPs@CVP Gel Microneedles

CMCS-Va gel was first prepared: 2.0 g of CMCS was dissolved in 30 mL of deionized water and stirred at room temperature for 30 min to form a uniform suspension; 1.0 g of Va was dissolved in 30 mL of deionized water, heated to 60 °C, and continuously stirred for 0.5 h [25,33]. CMCS and Va solutions were mixed at volume ratios of 1:0.5, 1:0.75, 1:1, 1:1.25, and 1:5 to prepare composite gels, which were then used for preliminary molding performance screening.

To improve the mechanical strength and molding properties of gel microneedles, PVA was incorporated to construct a CVP composite **gel** matrix. CMCS-Va and PVA were mixed at different mass ratios, and a predetermined amount of optimally synthesized AgNPs was added to the mixture, followed by the addition of Va solution to form a uniform drug-loaded gel precursor. The precursor was immediately transferred into a microneedle mould (A176, Taizhou Microchip Medical Technology, Taizhou, China), with air bubbles removed by vacuum and ultrasonication; centrifugation was used to drive the gel to the needle tip region. The mould was then vacuum-dried at 30 °C for 6 h, and the CVP gel microneedles were demoulded and stored in sealed bags at 4 °C for subsequent experiments.

The morphology was observed using digital cameras, microscopes, and SEM, while elemental compositions were analyzed by EDS and XPS.

### 4.4. Structural and Performance Characterization of AgNPs and AgNPs@CVP

Fourier transform infrared (FT-IR, VERTEX 70, Bruker) spectroscopy was used to identify chemical bonds and intermolecular interactions; ultraviolet-visible (UV-Vis, UV-6100S, Yuanxi Instruments, Shanghai, China) spectroscopy was used to verify the synthesis of AgNPs via surface plasmon resonance (SPR) characteristic peaks. A zeta potential analyser (90Plus PALS, Bruker) was used to determine the zeta potential of AgNPs for evaluating dispersion stability. A universal tensile testing machine and electronic universal testing machine were used to determine the tensile stress-strain and stress-displacement curves of the CVP gel matrix, and a sealing film penetration test was performed to evaluate the practical transdermal performance of the microneedles.

### 4.5. Evaluation of Biocompatibility and Migration Capability

Cytotoxicity Assay: L929 mouse fibroblasts were cultured in Dulbecco’s Modified Eagle Medium (DMEM) supplemented with 10% fetal bovine serum (FBS) and 1% penicillin-streptomycin, and maintained in a humid incubator at 37 °C with 5% CO_2_. When the cells reached 80% confluence, they were digested with 0.25% trypsin-EDTA, then subcultured and seeded at a density of 1 × 10^4^ cells per well in a 96-well plate, and incubated for 24 h to ensure complete cell adhesion [10]. The medium was then aspirated and replaced with fresh DMEM supplemented with AgNPs or AgNPs@CVP at gradient dilutions, with final concentrations of 25,50, and 100 μg/mL, respectively. Continue incubation for 24 h and 48 h separately. After incubation, add 10 μL of CCK-8 reagent to each well and incubate for 2 h under the same conditions. Subsequently, measure the absorbance at 450 nm in each well using an enzyme-labeled instrument [11]. Calculate cell viability using the blank control group cells treated with medium only without exposure to samples as the reference.

(1) $$\mathrm{Cell}\;\mathrm{Viability}=\frac{\mathrm{Sample}\;\mathrm{group}\;\mathrm{absorbance}-\mathrm{CCK}\text{-}8\;\mathrm{blank}}{\mathrm{Control}\;\mathrm{group}\;\mathrm{absorbance}-\mathrm{CCK}\text{-}8\;\mathrm{blank}}\;\times \;100\%$$

Live/Dead Cell Staining: L929 mouse fibroblasts were seeded at a density of 5 × 10^5^ cells per well in a 6-well plate and incubated overnight to ensure complete adhesion. The cells were then transferred to fresh DMEM medium containing AgNPs or AgNPs@CVP and co-incubated for 24 and 48 h, respectively. After incubation, the medium was aspirated, and the cells were gently rinsed twice with pre-cooled phosphate-buffered saline (PBS) to remove non-adherent cells and residual samples. Subsequently, the cells were stained for 15 min under light-shielded conditions at 37 °C using the Calcein AM/PI live/dead cell fluorescence staining kit. Finally, live cells (green fluorescence) and dead cells (red fluorescence) were observed and imaged using confocal laser scanning microscopy (CLSM) [39]. This experiment was designed to further validate the cytotoxic characteristics of the samples.

Cell Scratch Assay: L929 mouse fibroblasts were seeded into 6-well plates at a density of 2 × 10^5^ cells per well, and incubated until cellular confluence reached roughly 90%, at which point a continuous and intact cell monolayer was established. Using a sterile 200 μL pipette tip, draw a uniform linear scratch across the cell monolayer in each well; the detached cells and cellular debris were then thoroughly rinsed away with pre-warmed PBS (pH 7.4) to avoid interference with migration quantification. Subsequently, the culture medium was replaced with serum-free DMEM medium containing AgNPs or AgNPs@CVP. The plates were incubated under standard culture conditions (37 °C, 5% CO_2_), and bright-field images of the scratch area were captured at 0, 12, 24, 36, and 48 h using an optical microscope. The same visual fields were selected for imaging at each time point to quantitatively and qualitatively evaluate the cell migration-promoting capacity of the samples.

### 4.6. Antibacterial Activity Assay

Bacterial Culture: *E. coli*, *S. aureus*, *T-Salmonella*, and *C. albicans* were selected as the test pathogens. All strains were cultured on LB solid medium (4.0 g peptone, 2.5 g yeast extract, 5.0 g NaCl, 15.0 g agar, 500 mL deionized water, autoclaved at 121 °C for 0.5 h) at 37 °C for 12 h. Single colonies were picked and inoculated into LB liquid medium, shaken at 37 °C for 12 h, and the bacterial suspension was adjusted to a concentration of 1 × 10^7^ CFU/mL with deionized water for subsequent antibacterial experiments.

Antibacterial performance test detection: The agar diffusion assay was employed for the qualitative evaluation of the broad-spectrum antibacterial activity of the samples. Briefly, 400 μL of bacterial suspension (5 × 10^7^ CFU/mL) was uniformly spread onto LB solid medium plates using a sterile L-shaped spreader. Sterile filter paper discs (6 mm in diameter) were placed on the surface of the inoculated medium, and 6 μL of each test sample, including AgNPs, AgNPs@CVP and blank matrix controls (CMCS, Va, PVA), was added dropwise to the respective discs. A blank control group (PBS) was also established in parallel. All plates were incubated at 37 °C for 12 h in a humidified incubator. After incubation, the diameter of the inhibition zone surrounding each disc was measured using a vernier caliper (to the nearest 0.1 mm), and representative images of the plates were captured. Each experiment was performed in triplicate to ensure data reliability.

The microdilution method in 96-well plates was used to determine the MIC of the samples. 100 μL of sterile LB liquid medium was added to wells 2–12 of the 96-well plate, and 200 μL of the sample solution was added to well 1; two-fold serial dilution was performed from well 1 to well 12, and 100 μL of the solution in well 12 was discarded after mixing. 100 μL of bacterial suspension (1 × 10^7^ CFU/mL) was added to each well, with positive control wells (bacterial suspension + medium) and blank control wells (medium only) set up. The plates were incubated at 37 °C for 24 h, and the lowest sample concentration with no visible bacterial growth was defined as the MIC. For MBC determination, the sample solutions at the MIC and adjacent concentrations were pipetted onto LB solid medium plates, and 5 μL of each solution was added dropwise to three replicate points on the plate. Negative and positive controls were set up simultaneously, and the plates were incubated at 37 °C overnight. The lowest sample concentration with no bacterial colony growth on the plate was defined as the MBC.

The colony count experiment involved mixing AgNPs@CVP with bacterial suspension, followed by sampling at time points of 0, 5, 10, 20, 40, and 60 min. A 10 μL aliquot of bacterial suspension was inoculated onto solid agar medium and evenly spread, then incubated in a 37 °C incubator for 12 h before observation.

Antibacterial Mechanism Investigation: Bacterial suspension (1 × 10^8^ CFU/mL) was co-cultured with AgNPs or AgNPs@CVP at 37 °C for 12 h, then centrifuged at 5000 rpm for 5 min to collect the bacterial precipitate. The bacteria were stained with PI staining solution for 15 min, centrifuged to remove the supernatant, and washed three times with PBS. A 5 μL drop of the bacterial suspension was placed on a glass slide, and the red fluorescence was observed and imaged under a fluorescence microscope [40]. The blank control group was set up for comparison.

Bacterial suspension (1 × 10^8^ CFU/mL) was added to each well of a 96-well plate (200 μL per well) and incubated at 37 °C for 24 h under static conditions to form mature biofilms. After carefully collecting the culture supernatant, gently rinse each well three times with preheated PBS (pH 7.4) to completely remove unattached planktonic bacteria. Subsequently, 200 μL of AgNPs or AgNPs@CVP extract was added to the corresponding wells, followed by incubation at 37 °C for another 30 min; the blank control group was treated with an equal volume of PBS only. After incubation, the supernatant was discarded, and each well was fixed with 100 μL of methanol for 15 min at room temperature [41]. The methanol was then removed, and 50 μL of 0.1% (*w*/*v*) crystal violet staining solution was added to each well, followed by incubation in the dark for 30 min at room temperature. Excess unbound dye was removed by gently washing the plate three times with PBS, and the plate was air-dried at room temperature. Finally, 200 μL of absolute ethanol was added to each well to dissolve the crystal violet bound to the biofilm matrix, and the absorbance of each well at 590 nm was measured using a microplate reader [42]. The biofilm inhibition rate was calculated according to the following formula:

(2) $$\mathrm{Biofilm}\;\mathrm{Inhibition}\;\mathrm{Rate}\;\left(\%\right)=1-\frac{\mathrm{OD}(\mathrm{control}\;\mathrm{group})\;-\;\mathrm{OD}(\mathrm{experimental}\;\mathrm{group})}{\mathrm{OD}(\mathrm{control}\;\mathrm{group})}\times 100\%$$

where OD control and OD experimental represent the absorbance values of the blank control and sample groups, respectively.

### 4.7. Blood Compatibility and Animal Wound Healing Experiment

Haemolysis Assay: Orbital blood was collected from C57 mice and transferred to heparinized blood collection tubes. 1 mL PBS (pH 7.4) was added and gently mixed to prevent erythrocyte aggregation. The mixture was centrifuged at 1000 rpm for 10 min at 4 °C. The supernatant was carefully discarded, and the isolated red blood cells (RBCs) were washed three times with pre-cooled PBS to remove residual plasma components, yielding a homogeneous erythrocyte suspension (2% *v*/*v*) AgNPs and AgNPs@CVP were dispersed in PBS to prepare samples with final concentrations of 25, 50, and 100 μg/mL. Take 1 mL of each sample and mix it with an equal volume (1 mL) of erythrocyte suspension, then incubate in a 37 °C constant-temperature water bath for 1 h. After incubation, centrifuge the mixture at 3000 rpm for 10 min at 4 °C to precipitate intact erythrocytes, and measure the absorbance of the supernatant at 540 nm using an enzyme-labeled instrument. PBS and deionized water were used as negative control (no hemolysis) and positive control (complete hemolysis), respectively [43]. The hemolysis rate was calculated using the following formula:

(3) $$\mathrm{Hemolysisratio}\;(\%)=\frac{\mathrm{As}-\mathrm{An}}{\mathrm{Ap}-\mathrm{An}}\;\times \;100\%$$

The absorbance values of As, An and Ap were respectively represented by the sample, negative control and positive control.

Animal Model Establishment: Seventy-five male Sprague-Dawley (SD) rats (200–220 g) were acclimatized for 1 week under specific pathogen-free (SPF) conditions (25 ± 2 °C, 50 ± 5% relative humidity, 12 h light/dark cycle) prior to the experiment to minimize stress-induced variability. The rats were randomly assigned to five groups (*n* = 15 per group), with each group further stratified into normal full-thickness skin wound models and *S. aureus*-infected full-thickness skin wound models: blank control group (NC/IC), low-dose AgNPs@CVP gel microneedle group (NL/IL, 25 μg/mL), medium-dose group (NM/IM, 50 μg/mL), and high-dose group (NH/IH, 100 μg/mL). All animal experimental protocols were approved by the Research Ethics Committee of Shaanxi University of Technology (Approval No: 2025120901) and strictly performed in accordance with the Guide for the Care and Use of Laboratory Animals.

Prior to surgery, rats were anesthetized via inhalation of 3–5% isoflurane for induction and 1.5–2% isoflurane for maintenance (with oxygen as the carrier gas). The dorsal skin was carefully shaved using electric clippers, depilated with a commercial depilatory cream, and disinfected sequentially with 75% (*v*/*v*) ethanol and sterile 0.9% saline to eliminate microbial contamination. Two full-thickness circular wounds (8 mm in diameter) were created on the dorsal midline of each rat using a sterile punch biopsy, with a minimum distance of 2 cm between the two wounds to avoid cross-interference. In the infectious wound model, 100 μL of *S. aureus* suspension (1 × 10^8^ CFU/mL) was uniformly inoculated onto the wound surface immediately after injury and gently spread to ensure complete coverage of the defect area. Subsequently, AgNPs@CVP gel microneedles were directly applied to the wounds of each treatment group, while the blank control group Addedreceived no microneedle treatment [41,44]. All wounds were covered with sterile, breathable adhesive dressings to maintain a moist healing environment, and the microneedles were replaced at the same time daily throughout the experiment.

Wound Healing Evaluation: Digital photographs of the wounds were captured on days 0, 1, 3, 5, 7, and 9 post-wounding. The wound area was quantified using ImageJ software v1.53 to calculate the residual wound area ratio and wound closure rate. On days 3, 5, and 9 post-wounding, three rats were randomly selected from each group and euthanized humanely. Wound tissue samples (15 mm in diameter) were harvested, homogenized in phosphate-buffered saline (PBS), and centrifuged at 3000 rpm for 10 min at 4 °C. The resulting supernatant was collected to determine the expression levels of pro-inflammatory cytokines (TNF-α, IL-6) using commercial ELISA kits, strictly following the manufacturer’s protocols [45].

Histological evaluation: On days 5 and 9 post-wounding, wound tissue samples were collected from another three rats per group. The tissues were fixed in 4% paraformaldehyde for 24 h, dehydrated through a graded ethanol series, embedded in paraffin wax, and sectioned into 5 μm-thick slices using a microtome. The key histological features of wound repair, including epidermal hyperplasia, inflammatory cell infiltration, hemorrhage, edema, and capillary neovascularization, were observed through H&E staining and Masson staining [46]. Stained tissue sections were imaged under a brightfield microscope, followed by qualitative pathological analysis for evaluation.

### 4.8. Statistical Analysis

All experiments were conducted in triplicate, with data presented as mean ± standard error (SE). Statistical analysis was performed using IBM SPSS Statistics 25 software.

## Data Availability

All data are included in this manuscript.

## Supplementary Materials

The following supporting information can be downloaded at: https://www.mdpi.com/article/10.3390/gels12080736/s1, Figure S1: Optimization and Characterization of AgNPs; Table S1: Response surface test results; Table S2: AgNPs distribution map and total spectrum; Figure S2: Screening of microneedle matrices. Figure S3: Characterization and Performance Evaluation of AgNPs@CVP; Table S3: Drug-loaded microneedles distribution map and total spectrum; Table S4: MIC & MBC Comparison Table; Figure S4: Live images of antibacterial effects; Figure S5: Colony count images of drug-loaded microneedles; Figure S6: Hemolytic analysis chart.
