# Supplementary material for: Bifunctional Aloe-Birch Extracts for Green Silver Nanoparticle Synthesis and Synergistic Antimicrobial Microneedles Toward Infected Wound Healing
Source: Gels. 2026 Aug 17;12(8):736. doi: 10.3390/gels12080736 (PMC13512495; doi:10.3390/gels12080736)
Supplement: Supplementary file 1 [file gels-12-00736-s001.zip › gels-4474973-supplementary.pdf]

Supplementary

Bifunctional *Aloe-Birch* Extracts for Green Silver Nanoparticle Synthesis and Synergistic Antimicrobial Microneedles toward Infected Wound Healing

Shun Zhang [1], Xu Liu [1], Yiyun Wei [1], Kun Bao [1] Xiaojuan Zhang [1,2,3,*] and Chenlu Zhang [1,2,3,*]

1.School of Biological Science and Engineering, Shaanxi University of Technology, Hanzhong 723000, Shaanxi, China;
2.Shaanxi Province Key Laboratory of Bio-resources, Hanzhong 723000, Shaanxi, China; chenluzhang@snut.edu.cn
3.Qinba State Key Laboratory of Biological Resources and Ecological Environment (Incubation), Hanzhong 723000, Shaanxi, China

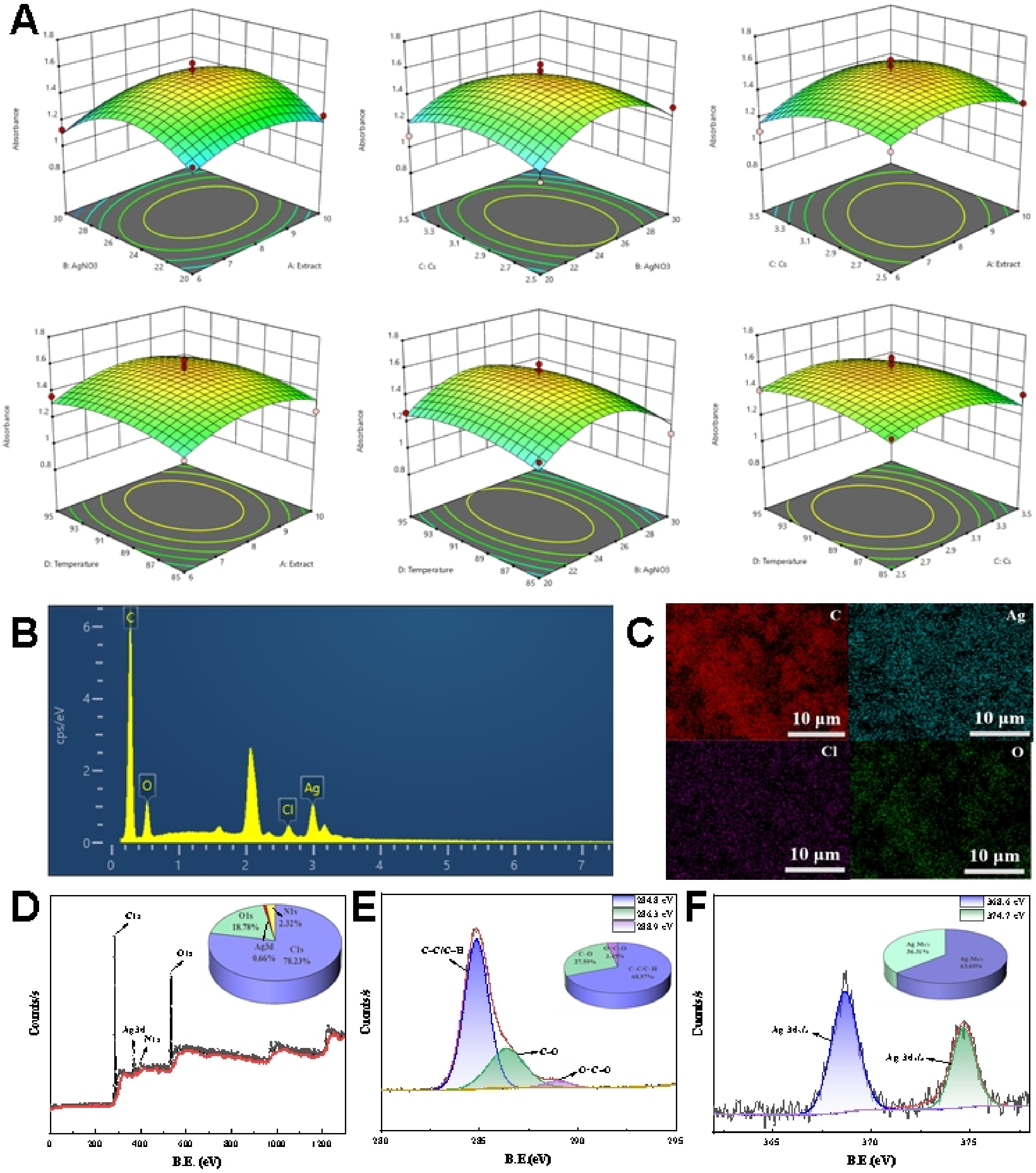

Figure. S1 Optimization and Characterization of AgNPs. (A) Coupled surface analysis diagram of response surface optimization; (B-C) EDS mapping of AgNPs; (D-F) XPS analysis spectrum of AgNPs.

Table. S1 Response surface test results.

Run	X1	X2	X3	X4	Y
1	10	25	3	85	1.2505
2	10	25	2.5	90	1.3097
3	8	25	2.5	95	1.3936
4	8	25	3	90	1.6303
5	8	25	3	90	1.5829
6	6	25	3	85	1.2505
7	8	20	3.5	90	1.0903
8	8	25	3	90	1.5205
9	6	25	3.5	90	1.0950
10	8	30	3	85	1.1148
11	8	25	2.5	85	1.3818
12	8	30	3	95	1.1201
13	10	20	3	90	1.2389
14	8	30	2.5	90	1.3097
15	8	20	3	95	1.2714
16	10	25	3	95	1.3668
17	8	20	2.5	90	1.1262
18	8	25	3	90	1.4639
19	8	25	3	90	1.4277
20	6	30	3	90	1.1300
21	6	25	3	95	1.3627
22	8	25	3.5	85	1.3599
23	10	25	3.5	90	1.3301
24	10	30	3	90	1.2589
25	8	20	3	85	1.2714
26	8	25	3.5	95	1.3668
27	6	20	3	90	1.2205
28	8	30	3.5	90	1.1300
29	6	25	2.5	90	1.3097

Table. S2 AgNPs distribution map and total spectrum.

element	Line type	wt%	Wt % Sigma	At%
C	K-series	58.24	0.35	81.95
O	K-series	11.99	0.21	12.66
Cl	K-series	2.25	0.10	1.07
Ag	L-series	27.52	0.36	4.31
total	/	100.00	/	100.00

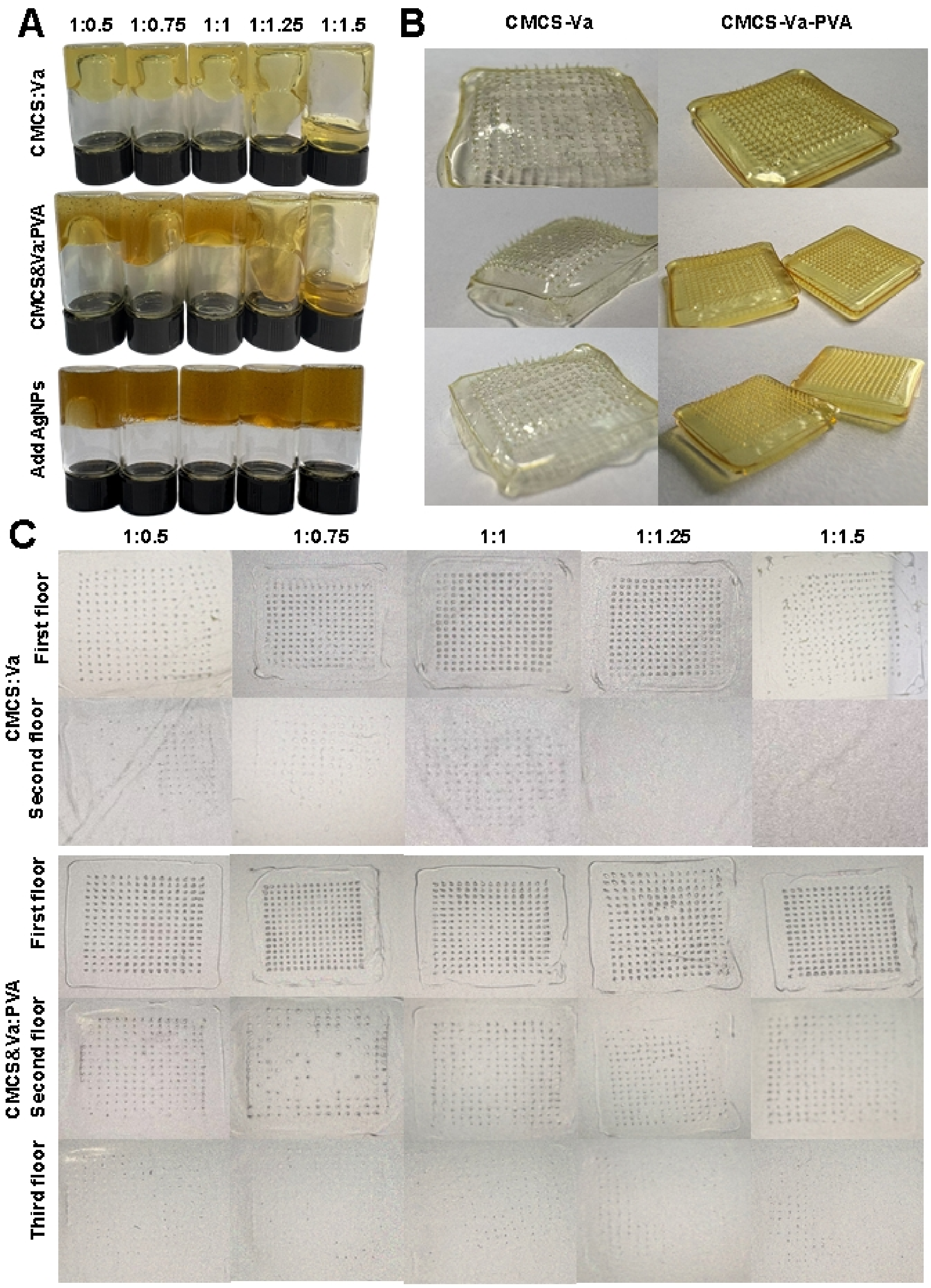

Figure. S2 Screening of microneedle matrices. (A-B) Real-time image comparison of microneedle formation and gelation processes prepared with different materials; (C) Penetration test of gel microneedle sealing films prepared with varying material ratios.

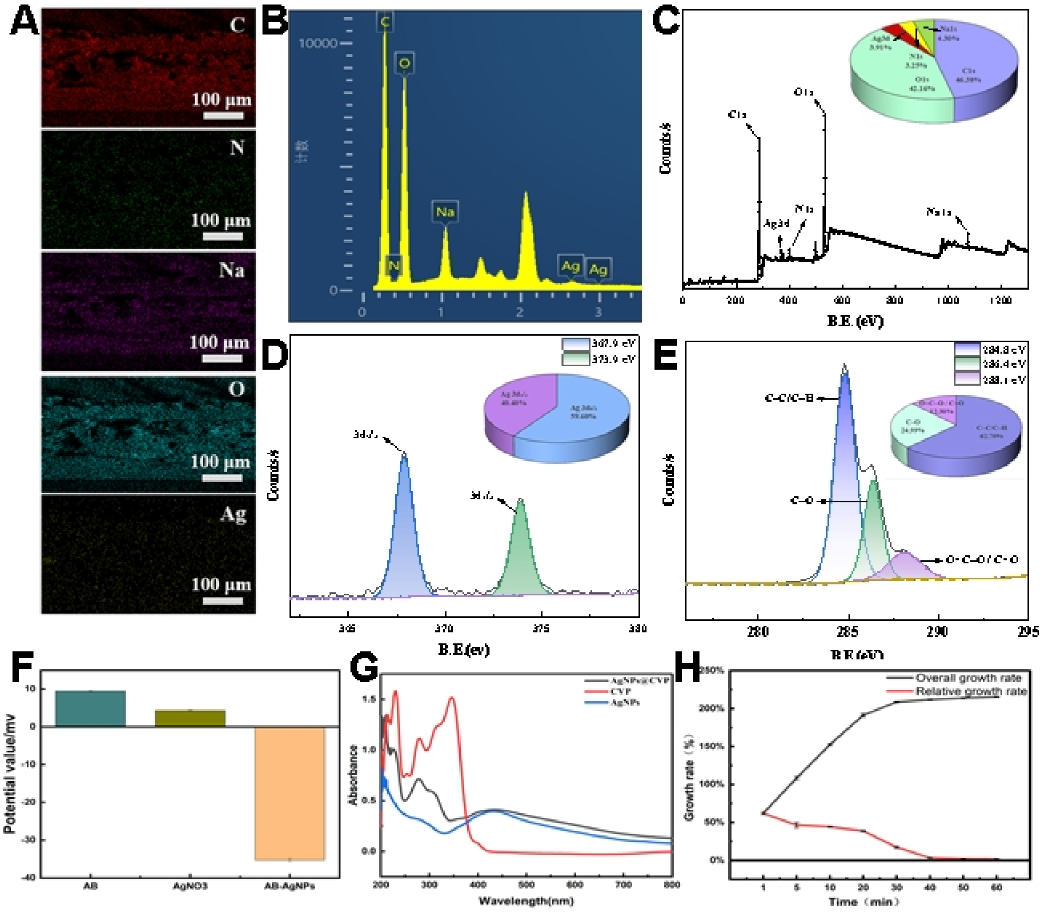

Figure. S3 Characterization and Performance Evaluation of AgNPs@CVP. (A-B) EDS mapping of AgNPs@CVP; (C-E) XPS spectrum of AgNPs@CVP; (F) Zeta potential changes during AgNPs formation; (G) UV-Vis absorption spectrum of AgNPs@CVP; (H) Swelling state curve of AgNPs@CVP microneedles.

Table. S3 Drug-loaded microneedles distribution map and total spectrum.

Element	Line type	wt%	Wt % Sigma	At%
C	K-series	54.53	0.46	62.23
N	K-series	2.03	0.71	1.99
O	K-series	38.26	0.36	32.78
Na	K-series	5.00	0.09	2.98
Ag	L-series	0.18	0.21	0.02
total	/	100.00	/	100.00

Table. S4 MIC & MBC Comparison Table.

		E. coli	*S. aureus*	*T-Salmonella*	*C. albicans*
M	AB	37.5mg/mL	15.625mg/mL	50mg/mL	15.625mg/mL
I	AB-AgNPs	23.438μg/mL	23.438μg/mL	31.25μg/mL	23.438μg/mL
C	AgNPs@CVP	3.906μg/mL	0.977μg/mL	1.953μg/mL	7.813μg/mL
M	AB	75mg/mL	25mg/mL	75mg/mL	31.25mg/mL
B	AB-AgNPs	39.063μg/mL	39.063μg/mL	78.125μg/mL	39.063μg/mL
C	AgNPs@CVP	15.625μg/mL	3.906μg/mL	15.125μg/mL	7.813μg/mL

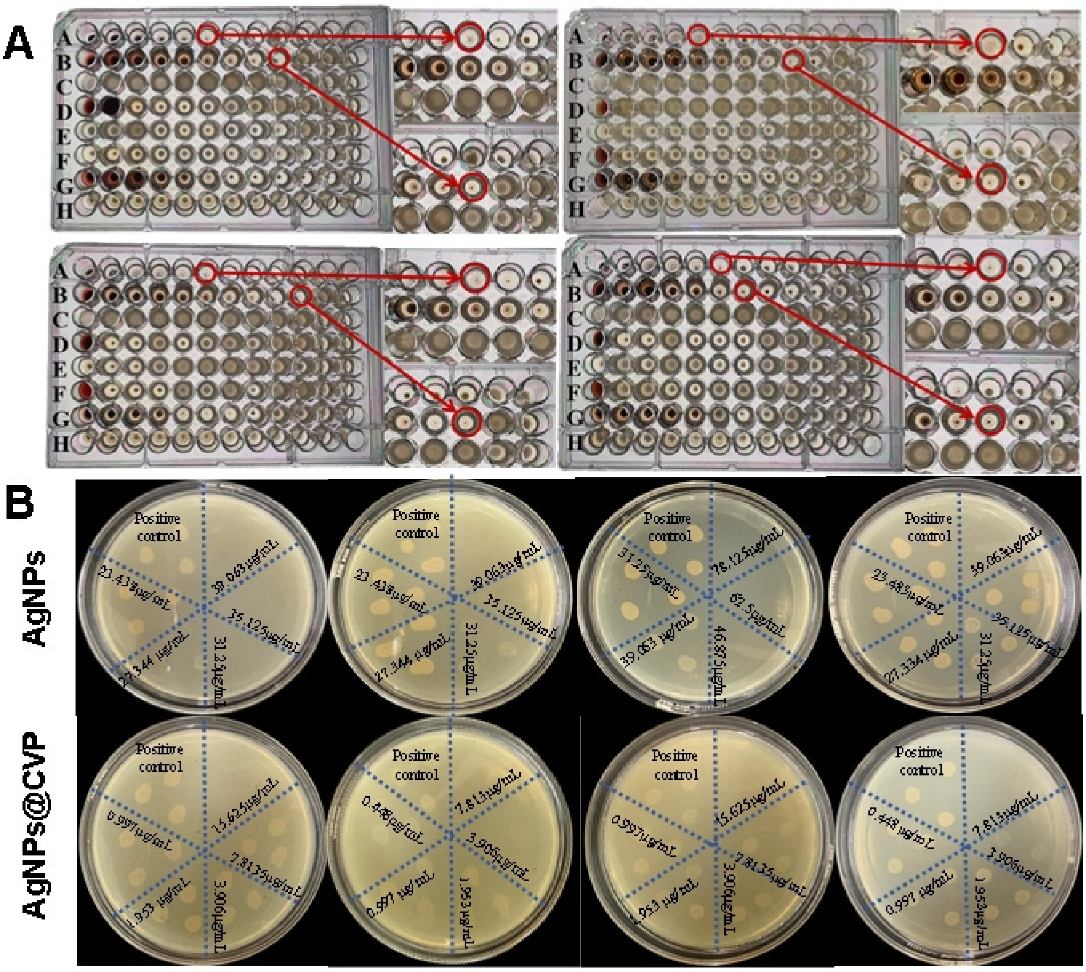

Figure. S4 Live images of antibacterial effects. (A) Real-time MIC plot of AgNPs@CVP; (B) Real-time MBC plot of AgNPs and AgNPs@CVP. A: AgNPs; B: AgNPs@CVP;C: CMCS; D:Va; E:CMCS -Va; F:PVA; G: CMCS -Va-PVA; H blank

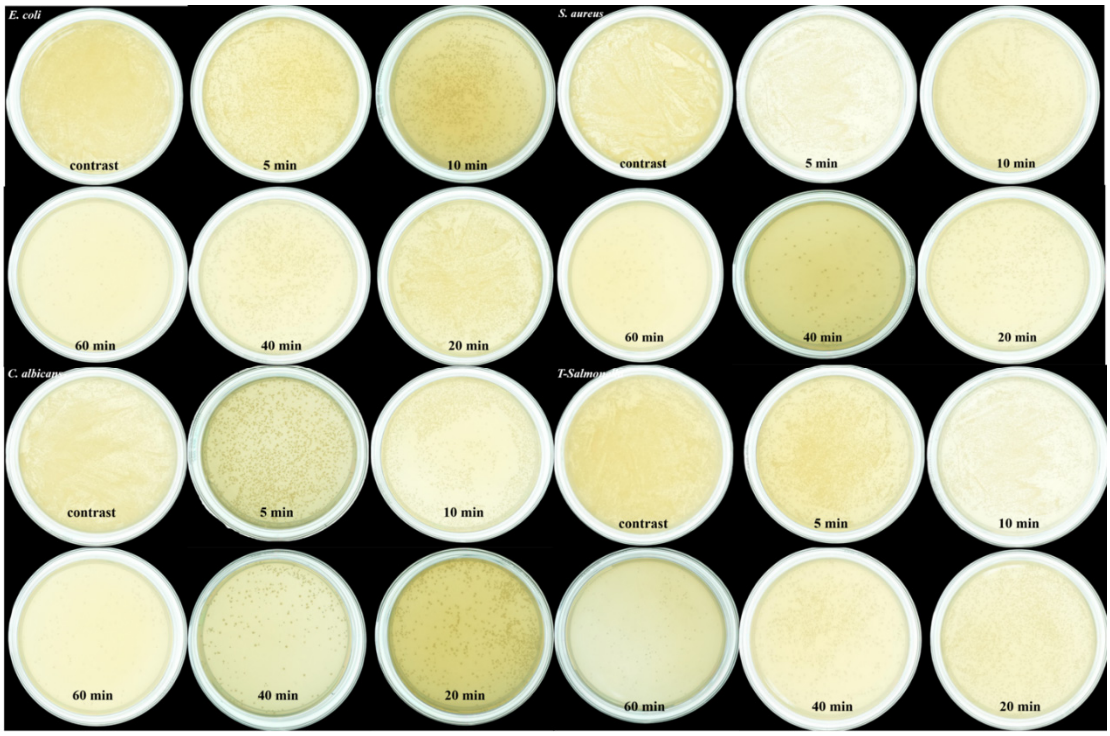

Figure. S5 Colony count images of drug-loaded microneedles.

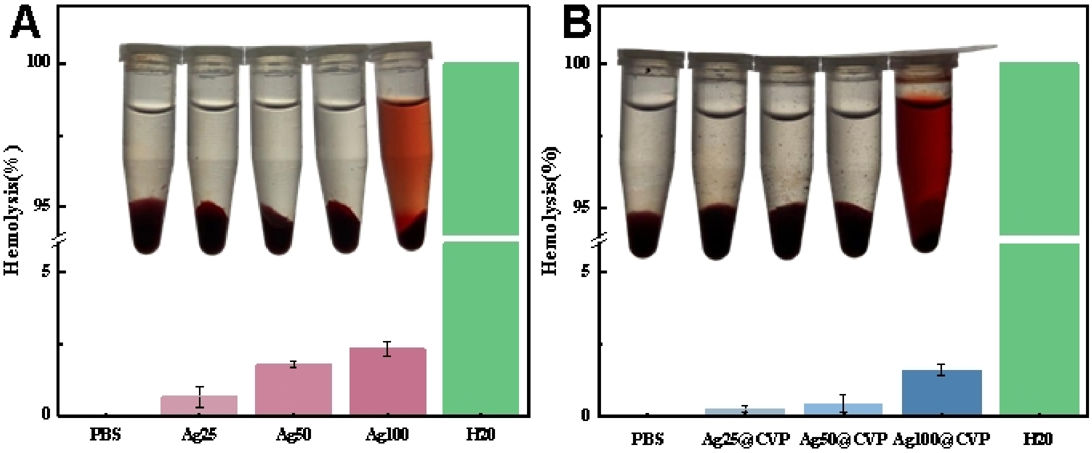

Figure. S6 Hemolytic analysis chart. (A) Hemolytic rate of AgNPs; (B) Hemolytic rate of AgNPs@CVP.
